# Mobile and Web-Based Apps That Support Self-Management and Transition in Young People With Chronic Illness: Systematic Review

**DOI:** 10.2196/13579

**Published:** 2019-11-20

**Authors:** Yisselle Ilene Virella Pérez, Sharon Medlow, Jane Ho, Katharine Steinbeck

**Affiliations:** 1 The Children’s Hospital at Westmead Academic Department of Adolescent Medicine Westmead, New South Wales Australia; 2 The University of Sydney, Faculty of Medicine and Health, Sydney Medical School Discipline of Child and Adolescent Health Westmead, New South Wales Australia; 3 Sydney Children’s Hospital Network, Sydney Children’s Hospital, Randwick, Centre for Adolescent and Young Adult Health Randwick, New South Wales Australia

**Keywords:** adolescent, mobile app, Web-based app, chronic illness, self-management, transition to adult care

## Abstract

**Background:**

More adolescents with chronic physical illness are living into adulthood, and they require the development of proficient self-management skills to maintain optimal physical health as they transition into adult care services. It is often during this vulnerable transition period that deterioration in illness control is seen as a result of inadequate self-management skills and understanding of their chronic illness. Mobile technology has been proposed as an innovative opportunity to assist in improving the management of chronic conditions as young people transition to adult care services. Over the past 5 years, there has been a significant increase in research into the use of health-related apps.

**Objective:**

This study aimed to evaluate the utility and effectiveness of mobile and Web-based health apps that support self-management and transition in young people with chronic physical health illnesses.

**Methods:**

We conducted a comprehensive review of the literature in 5 bibliographic databases, using key search terms, considering only articles published from 2013, as we were extending the data from 2 previous systematic reviews. Abstracts were screened for possible inclusion by 2 reviewers. Data extraction and quality assessment tools were used for the evaluation of included studies.

**Results:**

A total of 1737 records were identified from the combined electronic searches, and 854 records were removed as duplicates. A total of 68 full articles were further assessed for eligibility, and 6 articles met our review criteria: 3 pilot studies, 2 randomized controlled trials, and 1 prospective cohort study. Publication years ranged from 2015 to 2018. The apps reported were targeted at type 1 diabetes mellitus, epilepsy, asthma, beta thalassemia major, and sickle cell disease, with a combined sample size of 336. A total of 4 studies included in this review reported being effective in increasing knowledge of the targeted condition and increasing therapy adherence, including increased medication adherence. A total of 2 manuscripts only mentioned the word transition. Participant’s satisfaction was reported for all studies. Heterogeneity of the studies prevented meta-analysis.

**Conclusions:**

There remain limited data on the effectiveness and use of mobile and Web-based apps, which might facilitate the transition of adolescents with chronic illnesses from pediatric to adult health care services. This systematic review provides an updated overview of available apps for adolescents with chronic illnesses. This systematic review has been unable to provide evidence for effectiveness of this approach, but it does provide insights into future study design, with reference to the development, evaluation, and efficacy of apps tailored for adolescents with chronic illnesses, including the involvement of adolescents in such designs.

**Trial Registration:**

PROSPERO CRD42018104611; https://www.crd.york.ac.uk/prospero/display_record.php?RecordID=104611

## Introduction

With advances in medical science, more adolescents with chronic physical illness are now living into adulthood [1-3]. Chronic illnesses, by definition, are long-term health conditions that, if incorrectly managed or left untreated, are likely to have consequences for the overall well-being of the person [4]. Chronic illnesses have a substantial impact on health care systems, as patients are frequent users of health care resources, and they often require complex interventions and treatment to manage their condition over a lifetime [5]. The development of self-management skills is essential for adolescents living with chronic conditions to maintain optimal physical health as they take responsibility for their own health care. Barlow et al define self-management as “the individual's ability to manage the symptoms, treatment, physical and psychosocial consequences and lifestyle changes inherent in living with a chronic condition” [6]. Self-management skills are of particular importance for adolescents with a chronic illness as they transition into adult clinical care, with parents and caregivers no longer taking responsibility for these tasks and adult health care professionals expecting young people to come equipped with these skills [7].

Transition is defined as the planned movement of adolescents with chronic illness from pediatric-centered care to adult health care systems [8]. It is during this time that deterioration in illness control and even unplanned hospitalizations are often observed as a result of inadequate self-management and physical deterioration [9-12]. Therefore, it is important to find developmentally appropriate and accessible ways to encourage and promote the improvement of young people’s self-management behaviors [9].

Smartphones and tablets are widely used by adolescents and young adults. According to the Pew Research Center Internet and Technology (2018), an estimated 95% of adolescents in the United States own or have access to a smartphone, representing a 22% increase from 2014 to 2015. A total of 45% adolescents reported that they use the internet constantly on their device, nearly doubling the number from the 2014 to 2015 survey [13]. In the United Kingdom, 62% of adolescents aged 12 to 15 years own a mobile phone [14]. Similarly, 94% of Australians aged 16 to 17 years (age when active planning for the event of transfer should occur) owned a smartphone, and 89% of those between 18 and 24 years of age had a smartphone from which 83% of those downloaded an app [15], with numbers expected to continue rising. The multifunctional characteristics of smartphones and tablets allow multiple interventions and goals to be addressed, including knowledge enhancement and independent self-management skills [16]. It is not surprising that mobile and Web-based health technology has been proposed as a way to interact with young people with chronic illness as they enter the vulnerable transition period [8,17].

Currently, there are over 97,000 health-related apps available in the category of health and fitness in Google Play and the App Store, with approximately 1000 apps being created every month. It is estimated that the number of available apps will increase by about 25% each year [18]. Over the past 5 years, there has also been a significant increase in research on the utility of health-related apps [18].

To date, there has been little evidence produced about the use of mobile and Web-based apps aimed at supporting self-management in chronic illness and transition to adult care. A systematic review conducted by Majeed-Ariss et al [19] examined the literature published between 2003 and 2014 on the effectiveness of mobile apps designed to support adolescents’ management of their physical chronic or long-term conditions. A total of 4 studies were included in the systematic review. Cafazzo et al’s [20] and Frøisland et al’s [21] apps were targeted at type 1 diabetes. Cafazzo et al’s [20] pilot study trial showed that daily average frequency of blood glucose measurements increased by 50% (*P*=.006); however, glycated hemoglobin (HbA_1c_) did not change significantly (*P*=.11). On the contrary, Frøisland et al’s [21] study did not find any statistical significance in their outcomes, which were changes in HbA_1c_, system usability, and theoretical knowledge. Burbank et al developed an app to improve asthma management [22]. Participant’s satisfaction with the app was high (93%); asthma control tests scores improved significantly (*P*=.03), as well as asthma attack prevention self-efficacy scores (*P*=.04). Aldiss et al’s app was targeted at cancer [23]. Even though they included 6 psychometric measures, the authors did not identify a primary outcome measure. The authors of the systematic review were unable to draw any conclusions, primarily because of the fact that the studies included in the review were generally in early proof-of-concept phase and with few participants.

A recent meta-analysis drawing on data published between 2006 and 2016 examined the use of mobile health interventions in improving health outcomes in young people [24]. This study showed that digital technology can be effective in eliciting meaningful improvements in pediatric health behavior and associated health outcomes. However, the average age of participants was only 11.4 years, and many of the included studies focused on interventions targeting care givers, thereby limiting conclusions around adolescents with chronic illnesses. Some of the health behaviors addressed included immunization adherence, HIV prevention, dental hygiene, sun safety, physical activity, smoking, obesity, diabetes, stem cell transplant, and asthma.

In summary, the usefulness or effectiveness of mobile health technology interventions among adolescents remains unclear. Adolescents are more likely to use the newest and smartest technology, which makes the use of mobile apps more challenging. Technology evolves rapidly, and there is then the cost of competing with commercial developers who may be less interested in more niche markets [25], such as young people with chronic conditions.

The aims of this systematic review were the following: first, to evaluate the current available literature on the utility and effectiveness of mobile or Web-based health apps created for adolescents with chronic physical health conditions, which demonstrate some degree of user interaction over and above self-monitoring of the physical condition. Second, to describe features of the app that might explicitly or implicitly facilitate the transition of adolescents with chronic physical illness from pediatric to adult care.

## Methods

This systematic review was registered with the International Prospective Register of Systematic Reviews (PROSPERO): CRD42018104611.

### Search Strategy

A comprehensive literature search of 5 bibliographical databases was conducted to identify eligible studies. Databases included the following: Medical Literature Analysis and Retrieval System Online (MEDLINE), EMBASE, PsycINFO, Web of Science, and the Cumulative Index to Nursing and Allied Health Literature. The comprehensive search strategy was initially created in MEDLINE (Multimedia Appendix 1), and then it was adapted to the other databases. The specified chronic conditions were included to ensure that all available literature was included, and because these conditions require continuity of care and specialist-to-specialist transition. No restriction was placed on language. Only articles published from 2013 were considered, as this review is an update on the 2 previous systematic reviews cited in the Introduction section [19,24]. We also hypothesized that as general health-related app use research has increased in the past 5 years [18], this might also be reflected in an increase in adolescent studies. The end date for article searching was August 2018. Reference lists of relevant articles were hand searched to identify additional studies.

### Inclusion and Exclusion Criteria

Only original quantitative and qualitative studies published in peer-reviewed journals were included. Studies were required to focus on the use of a mobile device or Web-based app as an intervention to support self-management and to aid transition of adolescents diagnosed with a chronic physical illness from pediatric to adult clinical care. Outcomes included any changes in behavioral, physiological, attitudinal, or knowledge variables. Adolescents were young people aged 10 to 19 years, as defined by the World Health Organization [26].

Studies that included mobile or Web-based apps for the management of mental health, acute cancer, pain, or lifestyle/health risk behaviors were excluded. Chronic pain syndromes and mental health are undeniably important health issues for adolescents, but the management approaches have some significant differences to those of chronic physical illness. Management in chronic physical illness is around long-term and often complex regimens, which highlight differences to their peers and where adequate control is often difficult to ascertain on a day-to-day basis. The strategies are likely to be different in type or dose to those employed in mental health and chronic pain, where rehabilitation and reframing of perceptions and thought are key, making it difficult to extrapolate this information to chronic physical illness. Studies were also excluded if the app was used only for monitoring health status, such as continuous glucose monitoring for diabetes or if less than 50% of the sample size of the study was in the adolescent age range of 10 to 19 years. It is acknowledged that the timing of transition varies among countries, but a majority of adolescents with chronic illness will have commenced the transfer to adult health services by the time that secondary schooling ends.

### Study Selection

**Figure 1:**
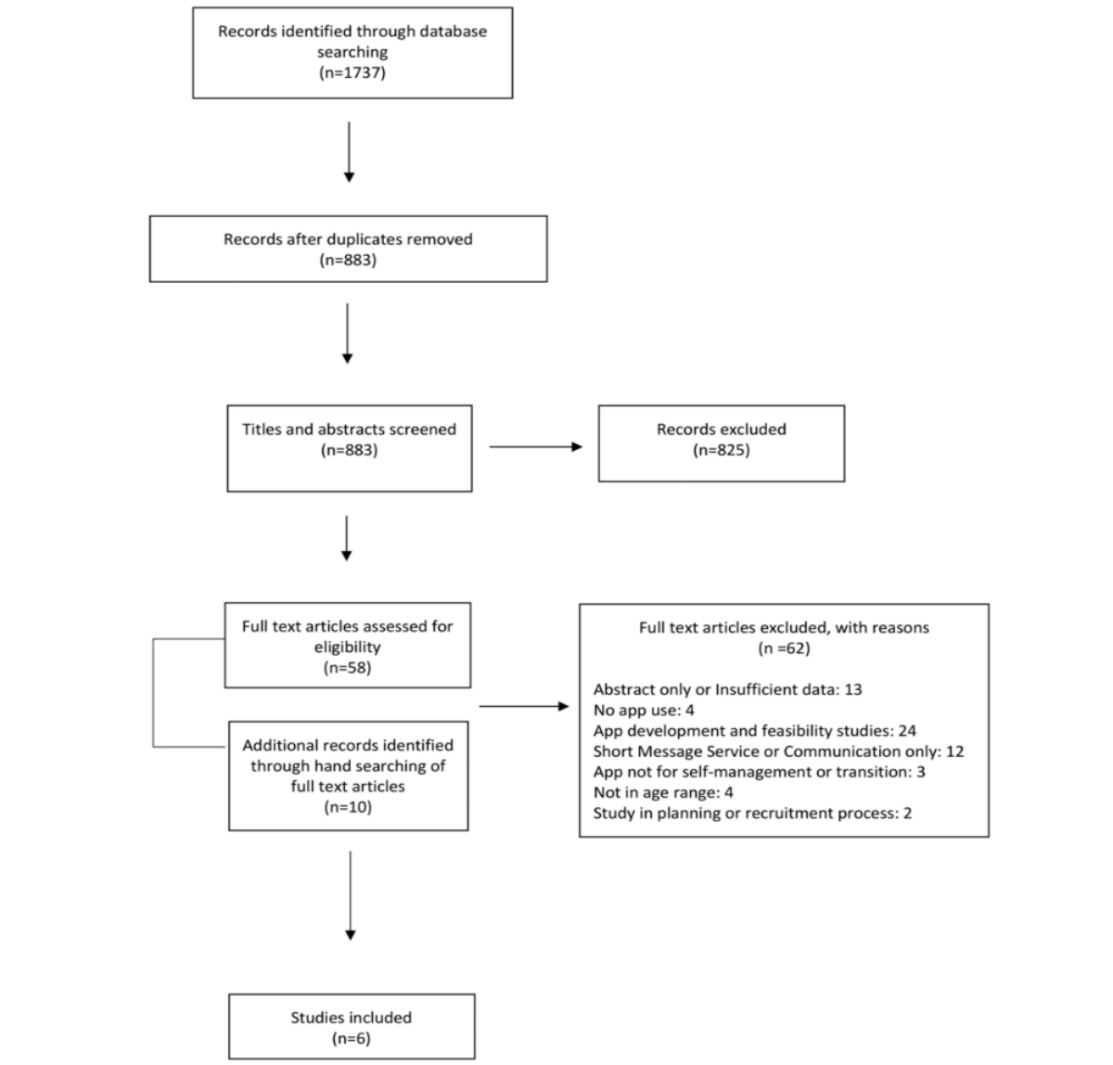
Preferred Reporting Items for Systematic Reviews and Meta-Analyses flow diagram of the review.

The design of this review followed the Preferred Reporting Items for Systematic Reviews and Meta-Analyses’ (PRISMA) statement [27], and the PRISMA diagram for study selection is shown in Figure 1. Duplicate studies were removed. A total of 1 reviewer (YVP) performed the first-stage screening of titles and abstracts on the basis of the research question, as well as the inclusion and exclusion criteria. Abstracts were then screened for possible inclusion by 2 reviewers (YVP and KS) to determine full-text screening. There were no disagreements between these 2 reviewers, but JH had been identified as the third party to resolve consensus issues. Scientific abstracts were considered if these were published in a peer-reviewed journal and contained adequate data to apply inclusion and exclusion criteria. A total of 2 corresponding authors were contacted via email to obtain further information to be included in the final review of manuscripts. As no responses were received, these 2 abstracts were excluded.

### Data Extraction

The information recorded included the following: demographics (age, gender, and country of origin), chronic physical illness, study characteristics (setting, study design, duration, data collection methodology, and sample size), and study outcomes. Any mention of transition was recorded. In addition, and when possible, further details of the app used to deliver an intervention were recorded: app name, platform, app’s purpose, content, education delivered about its use, availability (eg, was an access code required), as well as parent and health care professional access to the app.

### Quality Assessment

The checklist created by Downs and Black [28] for randomized and nonrandomized studies was used to assess the quality of the full manuscripts included in the systematic review. This scale is a 27-item checklist that assesses study reporting, external validity, internal validity in terms of bias and confounding (selection bias), and power. It has good reliability and high internal consistency [28]. The maximum score for the modified checklist was 28. Items were given 1 if the answer was “yes” or 0 if it was “no” or “unable to determine”. A total of 1 item (Are the distributions of principal confounders in each group of subjects to be compared clearly described?) had a maximum possible score of 2. The score of item 27 was modified, as has been done in previous studies [29,30], awarding 1 or 0 points depending on whether there was statistical power to detect a clinically important effect. Score ranges were grouped into 4 categories: excellent (26-28), good (20-25), fair (15-19), and poor (≤14), as previously defined by Samoocha et al [30].

### Statistical Analysis

Descriptive statistics were performed to capture the country where research was conducted, research setting, sample sizes, participant characteristics, health condition, study design, outcome measures, features, and usability of the apps. It was not possible to perform a meta-analysis because of the small number of manuscripts that met inclusion criteria and the heterogeneity of study outcomes.

## Results

The process of manuscript identification and selection is summarized in Figure 1. A total of 1737 records were identified from the combined electronic searches, from which 854 records were removed as duplicates. A total of 58 full-text articles, in addition to 10 articles identified through hand searching of full-text records’ bibliographies, were selected for full-text review, and 6 articles met inclusion criteria for data extraction.

### Characteristics of Included Studies

Characteristics of the 6 full manuscripts appear in Table 1.

Publication year ranged from 2015 to 2018. The studies describe 6 different apps used as interventions to support self-management or aid in transition of adolescents with different chronic physical illnesses: asthma [31,32], beta thalassemia major and sickle cell disease [33], type 1 diabetes mellitus [25,34], and epilepsy [16]. Of these manuscripts, 2 were pilot studies with an intervention group only [31,33], 1 was a pilot study with a control and intervention group [32], 2 were randomized controlled trials [25,34], and 1 was a prospective cohort study [16]. A total of 3 of the studies [25,32,34] compared 2 study groups, an intervention group in which the participants received usual care plus the intervention (app) and a control group that received only usual care; in 1 of these studies [32], the control group received a “control” version of the app to facilitate data transfer from an inhaler sensor. App features are summarized in Table 2. A total of 4 studies were conducted in North America, 1 in Australia and 1 in Denmark. All studies recruited participants from specialist clinics or pediatric hospitals. The setting details for the studies are outlined in Table 3. Sample sizes ranged from 7 to 151, with a combined sample size of 336. Participant ages ranged from 8 to 22 years, with a median of 14.1. The duration of the studies ranged from 1 to 12 months. There was an average 93% retention across studies.

**Table 1:** Characteristics of the included studies. The primary outcome measures were italicized for emphasis.

Author, Year	Country	Chronic condition	Initial/final	Duration (months)	Female/Male	Age (years), mean (range)	Study design	Outcome measures (*primary*)
Farooqui et al, 2015 [31]	United States	Asthma	24/21	1	9/12	11.6 (9-16)	Pilot Study	*MA ipod e-log*^a^, *PI*^b^*Survey*
Cushing et al, 2016 [32]	United States	Asthma	7/5	3	5/2	14.1 (11-18)	Pilot Study	*US Interview* or *FG*^c^, *RTMD^d^*
Goyal et al, 2017 [25]	Canada	Type 1 diabetes mellitus	92/91	12	51/41	14.1 (11-16)	Randomized controlled trial (RCT)	*HbA_1c_*, SMBG^e^, DQOLY^f^, DFRQ^g^, SCI^h^, RTCS^i^, 7-point LS ^j^, SS^k^ interview
Leonard et al, 2017 [33]	United States	βThal^l^ and SCD^m^	11/10	6	7/4	12.4 (8-21)	Pilot Study	*MA*^n^*self-videos*, *FQ*^o^; *KA*^p^
Castensøe -Seidenfaden et al, 2018 [34]	Denmark	Type 1 diabetes mellitus	151/148	12	81/70	17.6 (14-22)	RCT	*HbA_1c_*, PCD^q^, HCCQ^r^, PAID^s^
Le Marne et al, 2018 [16]	Australia	Epilepsy	51/36	2^t^	27/24	14.5 (13-19)	Prospective Cohort	*SKEQ*^u^*, AKEQ*^v^, SSES-C^w^, CATIS^x^, MA^y^:parent log, MARS^z^

^a^MA ipod e-log: medication adherence assessed through electronic logging in iPod Touch.

^b^PI Survey: postintervention survey, in house, no details available.

^c^US Interview or FG: Unstructured interview or focus group to discuss experience with inhaler and app, no details available.

^d^RTMD: Real Time Medication Data from the sensor.

^e^SMBG: Self-monitoring Blood Glucose.

^f^DQOLY: Diabetes Quality of Life for Youth Questionnaire, to measure quality of life.

^g^DFRQ: Diabetes Family Responsibility Questionnaire, to measure adolescent-guardian interaction around care.

^h^SCI: Self-Care Inventory, to measure adherence to treatment recommendations.

^i^RTCS: Readiness to Change Survey, to assess participant self-management.

^j^7-point LS: 7-point Likert Scale, to assess overall satisfaction with the “bant” app.

^k^SS interview: semistructured interview, in house, no details available.

^l^βThal: beta thalassemia major.

^m^SCD: sickle cell disease.

^n^MA self-reported videos: medication adherence self-reported videos “selfies.”

^o^FQ: Feasibility Questionnaire, to assess parents and participants feasibility, adherence, satisfaction, and ease of use of the Intensive Training Program mobile app.

^p^KA: Knowledge Assessment, to assess understanding of educational material presented in the modules.

^q^PCD: Perceived Competence in Diabetes, assesses patients’ experience of being able to manage diabetes successfully.

^r^HCCQ: Health Care Climate Questionnaire, assesses the degree to which patients perceived their health care providers as supporting their autonomy.

^s^PAID: Problem Areas in Diabetes, assesses diabetes-related distress.

^t^2: mean duration 70 days (SD 39.9).

^u^SKEQ: Self Knowledge of Epilepsy Questionnaire modified version.

^v^AKEQ: Adolescent Knowledge of Epilepsy Questionnaire, assesses general epilepsy knowledge.

^w^SSES-C: Seizure Self-Efficacy Scale for Children and Adolescents with Epilepsy, measures self-reported efficacy in managing patient’s own seizure disorder.

^x^CATIS: Child Attitude Toward Illness Scale, measures attitudes and feelings about having epilepsy.

^y^MA parent log: medication adherence logged by the parent or caregiver.

^z^MARS: Mobile Application Rating Scale, survey to collect feedback about the app.

**Table 2:** Features and usability of the apps.

Author, year	App name	App training	App content	Platform	App use
Farooqui et al, 2015 [31]	AsthmaCare	NR^a^	Daily reminders (medication use and personalized triggered avoidance strategies), interactive asthma treatment plan, and gamification features	AP^b^/ iTouch	100% of the participants used it once daily, with 81% of the participants using it multiples times a day for a period of 30 days
Cushing et al, 2016 [32]	Asthmahero	NR	Medication usage and reminders, medication adherence graphics	AP mobile/tablet	NR
Goyal et al, 2017 [25]	“bant**”**	1-hour tutorial	Automatic Data Transfer, Electronic Logbook, Trends, Trend Wizard, Reward System, banter, and Personal Health Record	AP/mobile	35% of the participants had a moderate^c^ or high use
Leonard et al, 2017 [33]	ITP^d^ app	Day 0 of a 90-day program	“real time” adherence tracking, education modules, and patient support (reminder alert messages and behavioral reinforcement)	AP mobile/tablet	81% of the participants used it daily for a period of 90 days
Castensøe -Seidenfaden et al, 2018 [34]	Young with Diabetes	10-min tutorial	My Page, My Department, Chat Room, Carbohydrate Counting, Information About, Tips Package, To Parents, and Reminder Function	AP and AN^e^ mobile/tablet	70% of the participants used it for at least 5 days out of 64 days
Le Marne et al, 2018 [16]	EpApp	Download appointment	Patients’ epilepsy profiles, medication reminders, seizure diary, and personalized seizure statistics and graphs	AP and AN mobile/tablet	23% of the participants used it daily for 28 days

^a^NR: not recorded**.**

^b^AP: Apple.

^c^Moderate: data upload less than 3 of 7 days; High: data upload ≥3 of 7 days**.**

^d^ITP: Intensive Training Program app**.**

^e^AN: Android**.**

**Table 3:** Study outcomes.

Author, year	Setting	Mention of transition in manuscript	Significant outcomes^a^
Farooqui et al, 2015 [31]	Outpatient Clinic Ohio State University and Wexner Medical Center, United States	No	Increased treatment adherence (85%) and avoidance of asthma triggers (69%). All participants reported better knowledge of asthma after using the app.
Cushing et al, 2016 [32]	Mount Sinai Hospital Pediatric Outpatient Clinics (New York, United States)	No	Increased treatment adherence and asthma control confidence improved through the use of mobile app message reminders that led to changes in the medication use routine.
Goyal et al, 2017 [25]	The Hospital for Sick Children Toronto and University of Toronto, Canada	No	No significant differences: glycated hemoglobin, impact on self-management.
Leonard et al, 2017 [33]	Department of Pediatrics Duke University, North Carolina, United States	No	Increased treatment adherence (80%) and knowledge retention (96%). Clinically relevant decrease in serum ferritin at 6 months (*P*=.07).
Castensøe -Seidenfaden et al, 2018 [34]	Nordsjælland, Hervel, Roskilde, and Køge Hospitals and Steno Diabetes Center Copenhagen Denmark	Abstract, keywords, and Conclusions	No significant differences: glycated hemoglobin, impact on self-management.
Le Marne et al, 2018 [16]	The Sydney Children’s Hospitals Network at Sydney, Australia	Introduction and Discussion	Increased treatment adherence (*P*=.045). Increased knowledge (*P*≤.005). No significant improvement in seizure burden or psychosocial measures.

^a^Results were statistically significant if *P*≤.05.

### User Input Preapp Development

A total of 3 studies tested the preliminary app design and incorporated the adolescents’ feedback in the most recent design of the app [16,25,34]. The design principles for the “bant app” were derived from thematic analysis of interviews conducted with adolescents who had type 1 diabetes, as well as their parents [20]. A total of 20 adolescents aged 12 to 16 years, with type 1 diabetes, were then recruited for a 12-week evaluation phase. The pilot trial study showed that daily average frequency of blood glucose measurements increased by 50% (*P*=.006). HbA_1c_ did not change significantly (*P*=.11). Satisfaction with the “bant” app was high, with 88% of the participants stating that they would continue to use the app.

Workshops, mail panel, and feedbacks, including young people with type 1 diabetes, their parents, and health providers, were performed for the development of the “Young with Diabetes” app [35]. A feasibility study was conducted for 5 weeks among health care providers and young people. Participants found the app helpful, providing them with a range of self-management supports, such as the opportunity to write to their health care providers. They all reported that they would recommend the app to peers. Health care providers described the app as both intuitive to use and relevant to collaborating with young people with type 1 diabetes mellitus.

Le Marne et al obtained feedback for the “EpApp” on preliminary design concepts, planned features, usability, draft educational content, and potential app names through initial focus groups composed by adolescents with epilepsy and their parents, which was evaluated in a prospective cohort study [16].

### The Apps

**Figure 2:**
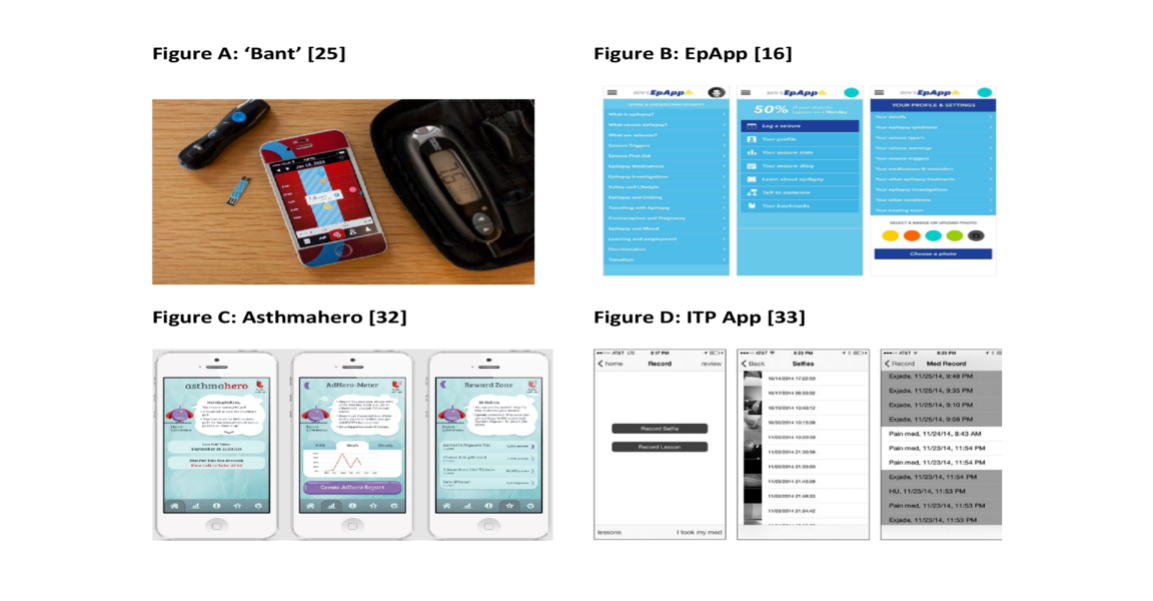
Screenshots of 4 apps.

A total of 4 of the included studies provided a screenshot of the mobile app used as an intervention, and these screen shots are reproduced as Figure 2. App features are summarized in Table 2.

Goyal et al [25] and Castensøe-Seidenfaden et al [34] used apps targeted at improving type 1 diabetes control. Le Marne et al’s app was for education and self-management of adolescents with epilepsy [16]. Cushing et al [32] and Farooqui et al [31] targeted asthma self-management skills, and Leonard et al addressed beta thalassemia major and sickle cell disease and chelation therapy [33].

The participants of 4 out of the 6 studies [16,25,33,34] had some app training before the use of the app (Table 2). Relevant concepts and medical terminologies [16], hardware setup, introduction to app features, username creation, troubleshooting step for potential issue [25], guidance session [34], and initial education module about iron overload and chelation therapy [33] were provided as part of this training.

Table 2 provides details on the interactive features of the different apps. All of the apps allowed participants to set medication reminders. A total of 5 apps came with a feature that graphically displays medication adherence [16,25,31,33,34], whereas one of the apps measured medication adherence through “selfie” video recording [33]. A total of 4 of the apps provided disease-related information [16,34,31,33], and 1 app also provided information on specific topics such as sex, alcohol, and holding a driver’s license having diabetes [34]. A total of 2 apps included a feature for social media interaction with peers [25,34]. The Intensive Training Program app used by Leonard et al allowed providers to send weekly messages to the patients, providing feedback and reinforcing the participants’ adherence behaviors [33]. A total of 3 of the apps provided rewards to encourage self-management behaviors [25,31,32]. The “bant” app participants [25] could redeem their points for iTunes gift cards, whereas the AsthmaCare [31] and Asthmahero [32] apps used gamification features with rewarding points to encourage medication adherence.

A total of 5 of the 6 studies reported frequency of app usage, but the grading of frequency varied among studies, as did the measurement chosen for usage (Table 2). For instance, Farooqui et al measured frequency in terms of electronic logging of medication use [31], whereas Leonard et al measured frequency in terms of data transfer (selfie videos) [33]. The grading of frequency also varied and generally relied on data upload frequency. Goyal et al reported the app use on the basis of the total number of days that the participants wirelessly uploaded blood glucose readings to the app over the 12 months [25]. They used 4 levels of engagement: very low, low, moderate, and high. “Young with Diabetes” use was defined as using the app for at least 5 out of 64 days [34]. On the contrary, Le Marne et al categorized app use as “daily,” “weekly,” “monthly,” “occasionally,” or “never” [16].

There were a variety of features relevant to convenience and privacy. For example, 2 studies [25,31] required the participant to carry a device other than the participant’s own smartphone (iPhone or iPod Touch). This was provided as part of the intervention. A total of 3 studies [16,33,34] allowed the participants’ parents to access the app, whereas 2 studies allowed health care professionals to [33,34] access the app. Only 2 studies mentioned the word transition [16,34]. Castensøe-Seidenfaden et al mentioned that “young people often struggle to self-manage type 1 diabetes during the transition from childhood to adulthood” and that “health care providers should routinely address sensitive topics and be aware of parents’ need for guidance as to how to effectively support their child during the transition from childhood to adulthood” [34]. Le Marne et al stated that “self-management tools have the potential to help scaffold adolescents as they transition to adulthood” and that “developing independence and responsibility for medication is an important step in transitioning from adolescence to adulthood” [16]. None of the included studies assessed effective transition from pediatric to adult health care services as an outcome.

A total of 1 study made a reference to cost, the app being freely available via Android and Apple platforms [16]. Technical specifications varied across the apps, as did reports of problems encountered. A total of 3 studies [16,33,34] provided an app access code that permitted analytics on app usage. A total of 3 studies reported on technical issues: faulty alerts on Android devices [16], unable to open the app (Apple), unable to upload photos (Android), app not opening because of update and reinstallation needed (Apple) [34], and video recording problems [33].

### Outcomes

Table 3 presents the outcome data for the 6 studies. Neither of the type 1 diabetes mellitus studies showed improvement in HbA_1c_ or self-management [25,34]. Exploratory analysis by Goyal et al showed that users who had more frequent self-monitoring of blood glucose had improvements in HbA_1c_ [25]. Castensøe-Seidenfaden et al concluded that the app might be a useful tool to complement self-management in adolescents with type 1 diabetes [34]. The other 4 studies reported increased knowledge of the condition and increased therapy adherence, including increased medication adherence through the use of “selfie videos” of the participant receiving daily chelation therapy [16,31-33].

Participants’ satisfaction was reported for all studies. A total of 3 quarters of the participants were very satisfied with the “bant” app [25] and 96% of the participants reported that they would continue to use the app if it were available to them outside of the trial. A total of 45% of the subjects ranked the trending feature, which reports consecutive out-of-range blood glucose readings and prompts the user to identify the likely cause and potential solution of the trend, as the most useful component of the app [25]. A total of 78% of the participants reported that the “Young with Diabetes” app was helpful, and 85 % of the participants reported they would recommend it to others [34]. The “chat room” and “my page” were the most popular features. Topics such as sex, diabetes, driver’s licenses, alcohol, and parties were the most retrieved from the information feature of the app [34]. Participants in the “EpApp” study identified themes relating to information content and reminders as the most helpful app features [16]. “Asthmahero” [32] helped as a medication reminder and improved the participants’ confidence about asthma control. The gaming aspects of the app served as motivation for treatment adherence. All “Asthmacare” users reported better understanding of their asthma after the intervention, and 95% of the participants preferred using an app for asthma education over other modalities [31]. Participants of the Intensive Training Program for chelation reported that the app was easy to use and endorsed the statement that continuous use of the app would be helpful for therapy adherence [33].

### Quality Assessment

The quality of the included full manuscripts varied, with individual scores shown in Multimedia Appendices 2 and 3. A total of 2 of the studies were rated as being “good,” 3 studies were rated to be “fair,” and 1 study was rated as “poor.” The mean score of the included studies was 17.3 (range 14-22). Studies scored poorly on the following quality assessment items: clearly describing the distribution of principal confounders (4/6), participant blinding (6/6), blinding of the outcome appraiser (6/6), subject randomization (4/6), and failure to adjust for confounding factors in the analysis (4/6). None of the studies described the entire participant population and as a consequence the external validity of all the studies was rated “poor.” Only 2 studies reported power to detect any clinically important effect [25,34].

## Discussion

### Principal Findings

The first aim of this systematic review was to evaluate the utility and effectiveness of mobile or Web-based health interventions targeting adolescents with chronic physical illnesses. Despite the proliferation of health apps over the past 5 years [18], our systematic review identified only 6 new studies. This may reflect the cost and smaller potential user base for what are quite complex apps. Few studies reached statistical significance in any of their multiple chosen outcome measures despite the relevance of these outcomes. Possible reasons for this finding include inadequate power, too short a duration of use, and failure to sufficiently engage adolescent users. A total of 2 studies specifically commented on a fixed design, which did not allow tailoring to adolescent needs [25,34]. Furthermore, when significant outcomes were reported, these related to adherence, except in diabetes, the most frequent specialist-to-specialist transition [36]. The latter may be because of the intrinsic utility of the app or because of inadequate duration of the study to influence the outcome measure of HbA_1c_.

The use of apps is promoted as having the potential to improve patterns of self-management support [37] that are necessary during the transition process to adult care. However, most studies identified in the initial searches focused on mobile or Web-based health interventions for adult self-management, rather than for adolescents. The data from adult studies likely have limited generalizability to adolescents. Adolescents have grown up in a technological era, fully embracing technology as a way to interact with others and becoming both expert and selective in electronic media and on social networking sites [38]. Apps developed for adults tend to be more directive [39,40], as opposed to delivering features that might engage young people [41]. Only 3 studies reported adolescents in predevelopment design, but it was difficult to assess if this codesign had an impact on outcomes.

The secondary aim of the study was to describe features of the apps that might explicitly or implicitly facilitate the transition of adolescents with chronic physical illness from pediatric to adult care. We were unable to draw any conclusions related to this aim. None of the studies measured transition process as an outcome; indeed, there are no established, validated successful outcome criteria for the transition process of adolescent and young adults with chronic illness [42]. In addition, the duration of most of the included studies was less than 6 months, which would not provide an adequate length of time to measure transition outcomes.

Education was a feature in 4 apps [16,31,33,34]. Educating the patient is an important aspect for the development of self-management skills. The ongoing accessibility of illness-specific educational themes in an electronic device allows patients to easily obtain reliable information. Nevertheless, education alone is insufficient to increase medication adherence and the development of self-management skills [9,43], and adding features, such as reminders, goal setting, rewards, and social media interaction with peers, may enhance better outcomes [43].

A total of 2 apps [25,34] included a feature that allowed the participants to share their experiences with other peers through social media to complement education and reassurance of common lived experiences. In addition, 2 apps [31,32] included gamification features, and 5 apps included graphical displays of illness-specific control measures. Patients liked the gaming aspects of these mobile apps and felt that earning points served as motivation for medication adherence and continuous app use. This feature would add considerably to future app development. Knowing what features patients like in the apps might assist in the development of a generic rather than an illness-specific app that could incorporate these identified common features and be easily tailored for purpose through simple “in-app” modifications.

No study presented an economic evaluation. Even though mobile health interventions are often described as cost-effective tools for patients living with chronic illnesses, there are limited economic data to support this [44]. A total of 2 systematic reviews have been conducted to assess the literature on the economic value of mobile health interventions. Both reviews concluded that there was a lack of economic data to support mobile health interventions for patients with chronic illness [45,46]. Iribarren et al suggested that studies should follow the established economic reporting guidelines to improve the available data [45], whereas Badawy and Kuhns highlighted the need for comprehensive economic evaluation of these interventions to understand the association between their cost-effectiveness while supporting self-management of patients with chronic illnesses [46].

A limitation of our review was the small number and heterogeneity of the studies that precluded meta-analysis. The lack of data about app development strategies that included adolescent input is an important limitation around feasibility and utility. The strength of this review is that it provides an up-to-date overview of apps as these relate to health care in adolescents with chronic illness. The small number of studies identified allowed us to describe apps in more detail, which may be helpful to those considering app development.

### Conclusions

In conclusion, this review has found that there remain limited data about the utility and effectiveness of mobile and Web-based apps facilitating self-management and the transition of adolescents with chronic illness from pediatric to adult health care services. The latter is a key time when independent adolescent-friendly assistance is required. This potentially reflects the lack of commercial appeal of such apps and the high cost of developing apps that compete with the thousands available to young people. Future studies could consider cocreation with adolescents, financial assistance from lay illness support groups, and easy but confidential ways to report feedback through the app.

## Appendix

Multimedia Appendix 1 Search strategy Medical Literature Analysis and Retrieval System Online via OvidSP (1946-present).

Multimedia Appendix 2 Description of items and scores used for the quality assessment of the included studies.

Multimedia Appendix 3 Designated values for the quality assessment of the included studies.

## Abbreviations

HbA_1c_: glycated hemoglobin
MEDLINE: Medical Literature Analysis and Retrieval System Online
PRISMA: Preferred Reporting Items for Systematic Reviews and Meta-Analyses
