# Supplementary material for: Mobile and Web-Based Apps That Support Self-Management and Transition in Young People With Chronic Illness: Systematic Review
Source: J Med Internet Res. 2019 Nov 20;21(11):e13579. doi: 10.2196/13579 (PMC6893564; doi:10.2196/13579)
Supplement: Multimedia Appendix 1 [file jmir_v21i11e13579_app1.pdf]

Multimedia Appendix 1: Search strategy Medline via OvidSP (1946-present).

1. adolescent/ or young adult/
2. adolescen* .tw
3. young adj2 (adult* or person* or people or man or men or male* or woman or women or female*) .tw
4. teen* .tw
5. youth* .tw
6. 1 or 2 or 3 or 4 or 5
7. Cell phone/
8. cell phone* .tw
9. exp Computers, Handheld/
10. smartphone* .tw
11. mobile adj (technology or phone* or application* or app or apps) .tw
12. app or apps .tw
13. Mobile Applications/
14. Web based application* .tw
15. 7 or 8 or 9 or 10 or 11 or 12 or 13 or 14
16. 6 and 15
17. Exp Chronic Disease
18. (chronic or persistent or long-term* or long standing) ajd2 (illness* or disease* or condition*) .tw
19. Cystic Fibrosis/
20. cystic fibrosis .tw
21. Diabetes Mellitus, Type 1/
22. diabetes .tw
23. Polycystic Ovary Syndrome/
24. polycystic ovar* .tw
25. Adrenal Hyperplasia, Congenital/
26. Congenital Adrenal Hyperplasia .tw
27. Klinefelter Syndrome/
28. Klinefelter* Syndrome .tw
29. Turner Syndrome/
30. Turner* Syndrome .tw
31. Cerebral Palsy/
32. cerebral palsy .tw
33. Epilepsy/
34. epilepsy* .tw
35. exp Neurofibromatoses/
36. neurofibromatos* .tw
37. Celiac Disease
38. coeliac .tw
39. celiac .tw
40. exp Inflammatory Bowel Diseases/
41. ulcerative colitis .tw
42. chron* Disease .tw
43. duodenal diseases/ or ileal diseases/ or jejunal diseases/
44. duodenal disease* .tw
45. jejunal disease* .tw
46. ileal disease* .tw
47. small bowel disease* .tw
48. hemophilia a/ or hemophilia b/
49. Haemophilia .tw
50. hemophilia .tw

51. exp Thalassemia/
52. Thalass* .tw
53. Anemia, Sickle Cell/
54. sickle* .tw
55. muscular dystrophies/ or muscular dystrophy, duchenne/ or myotonic dystrophy/
56. muscular dystroph* .tw
57. Duchenne* .tw
58. myotonic dystrophy .tw
59. Asthma/
60. asthma* .tw
61. Heart Defects, Congenital/
62. congenital heart* .tw
63. Arthritis, Juvenile/
64. arthritis .tw
65. rheumatoid adj (arthritis or disease*) .tw
66. scleroderma, localized/ or scleroderma, systemic/ or scleroderma, diffuse/ or scleroderma, limited/
67. scleroderm* .tw
68. Lupus Erythematosus, Systemic/
69. systemic lupus Erythematosus .tw
70. Ehlers-Danlos Syndrome/
71. ehlers danlos .tw
72. Marfan Syndrome/
73. Marfan* .tw
74. Phenylketonurias/
75. phenylketonuria .tw
76. Liver Cirrhosis/
77. cirrhosis .tw
78. Osteogenesis imperfecta/
79. osteogenesis imperfecta .tw
80. Kidney Diseases/
81. kidney disease* .tw
82. Spina Bifida/
83. spina bifida .tw
84. 17 or 18 or 19 or 20 or 21 or 22 or 23 or 24 or 25 or 26 or 27 or 28 or 29 or 30 or 31 or 32 or 33 or 34 or 35 or 36 or 37 or 38 or 39 or 40 or 41 or 42 or 43 or 44 or 45 or 46 or 47 or 48 or 49 or 50 or 51 or 52 or 53 or 54 or 55 or 56 or 57 or 58 or 59 or 60 or 61 or 62 or 63 or 64 or 65 or 66 or 67 or 68 or 69 or 70 or 71 or 72 or 73 or 74 or 75 or 76 or 77 or 78 or 79 or 80 or 81 or 82 or 83
85. 16 and 84
86. limit 85 to yr="2013 -Current"
87. patient transfer/ or transition to adult care/ or transitional care
88. transition* .tw
89. 87 or 88
90. 85 and 89
91. limit 90 to yr="2013 -Current"
92. Self-Management/
93. self management .tw
94. 92 or 93
95. 85 and 94
96. limit 95 to yr="2013 -Current"
97. 89 and 95
98. limit 97 to yr="2013 -Current"
