# Supplementary material for: Mobile and Web-Based Apps That Support Self-Management and Transition in Young People With Chronic Illness: Systematic Review
Source: J Med Internet Res. 2019 Nov 20;21(11):e13579. doi: 10.2196/13579 (PMC6893564; doi:10.2196/13579)
Supplement: Multimedia Appendix 2 [file jmir_v21i11e13579_app2.pdf]

Multimedia Appendix 2: Description of items and scores used for the quality assessment of the included studies.

Item Category	Item Code	Max Score	Description of quality assessment item
**Reporting**	A	1	Is the hypothesis/aim/objective of the study clearly described?
	B	1	Are the main outcomes to be measured clearly described in the Introduction or Methods section?
	C	1	Are the characteristics of the patients included in the study clearly described?
	D	1	Are the interventions of interest clearly described?
	E	2	Are the distributions of principal confounders in each group of subjects to be compared clearly described?
	F	1	Are the main findings of the study clearly described?
	G	1	Does the study provide estimates of the random variability in the data for the main outcomes?
	H	1	Have all important adverse events that may be a consequence of the intervention been reported?
	I	1	Have the characteristics of patients lost to follow-up been described?
	J	1	Have actual probability values been reported for the main outcomes except where the probability value is less than 0.001?
**External validity**	K	1	Were the subjects asked to participate in the study representative of the entire population from which they were recruited?
	L	1	Were those subjects who were prepared to participate representative of the entire population from which they were recruited?
	M	1	Were the staff, places, and facilities where the patients were treated, representative of the treatment the majority of patients received?
**Internal validity-bias**	N	1	Was an attempt made to blind study subjects to the intervention they have received?
	O	1	Was an attempt made to blind those measuring the main outcomes of the intervention?
	P	1	If any of the results of the study were based on "data dredging", was this made clear?
	Q	1	In trials and cohort studies, do the analyses adjust for different lengths of follow-up of patients, or in case-control studies, is the time period between the intervention and outcome the same for cases and controls?
	R	1	Were the statistical tests used to assess the main outcomes appropriately?
	S	1	Was compliance with the intervention/s reliable?
	T	1	Were the main outcome measures used accurate?
**Internal validity-confounding (selection bias)**	U	1	Were the patients in different intervention groups or were the cases and controls recruited from the same population?
	V	1	Were study subjects in different intervention groups or were the cases and controls recruited over the same period of time?
	W	1	Were the study subjects randomized to intervention groups?
	X	1	Was the randomized intervention assignment concealed from both patients and health care staff until recruitment was completed and irrevocable?
	Y	1	Was there adequate adjustment for confounding in the analyses from which the main findings were drawn?
	Z	1	Were losses of patients to follow-up taken into account?
**Power**	AA	1	Did the study have sufficient power to detect a clinically important effect where the probability value for a difference being due to chance is less than 5%?
