# Supplementary material for: Mobile and Web-Based Apps That Support Self-Management and Transition in Young People With Chronic Illness: Systematic Review
Source: J Med Internet Res. 2019 Nov 20;21(11):e13579. doi: 10.2196/13579 (PMC6893564; doi:10.2196/13579)
Supplement: Multimedia Appendix 3 [file jmir_v21i11e13579_app3.pdf]

Multimedia Appendix 3: Designated values for the quality assessment of the included studies.

Item code	A	B	C	D	E	F	G	H	I	J	K	L	M	N	O	P	Q	R	S	T	U	V	W	X	Y	Z	AA	STUDY SCORE
**Max Score**	**1**	**1**	**1**	**1**	**2**	**1**	**1**	**1**	**1**	**1**	**1**	**1**	**1**	**1**	**1**	**1**	**1**	**1**	**1**	**1**	**1**	**1**	**1**	**1**	**1**	**1**	**1**	**Max [28]**
Farooqui [31]	1	1	1	1	0	1	1	0	1	1	0	0	1	0	0	1	1	0	1	1	1	0	0	0	0	1	0	15
Cushing [32]	1	1	1	1	0	1	1	0	1	1	0	0	1	0	0	1	1	0	0	1	1	0	0	0	0	1	0	14
Goyal [25]	1	1	1	1	1	1	1	0	1	1	1	0	1	0	0	1	1	1	1	1	1	1	1	0	1	1	1	22
Leonard [33]	1	1	1	1	0	1	1	0	1	1	0	0	1	0	0	1	1	1	1	1	1	0	0	0	0	1	0	16
Castensoe-Seidenfaden [34]	1	1	1	1	1	1	1	0	1	1	0	0	1	0	0	1	1	1	1	1	0	1	1	0	1	1	1	20
Le Marne[16]	1	1	1	1	0	1	1	0	1	1	0	0	1	0	0	1	1	1	1	1	1	1	0	0	0	1	0	17
